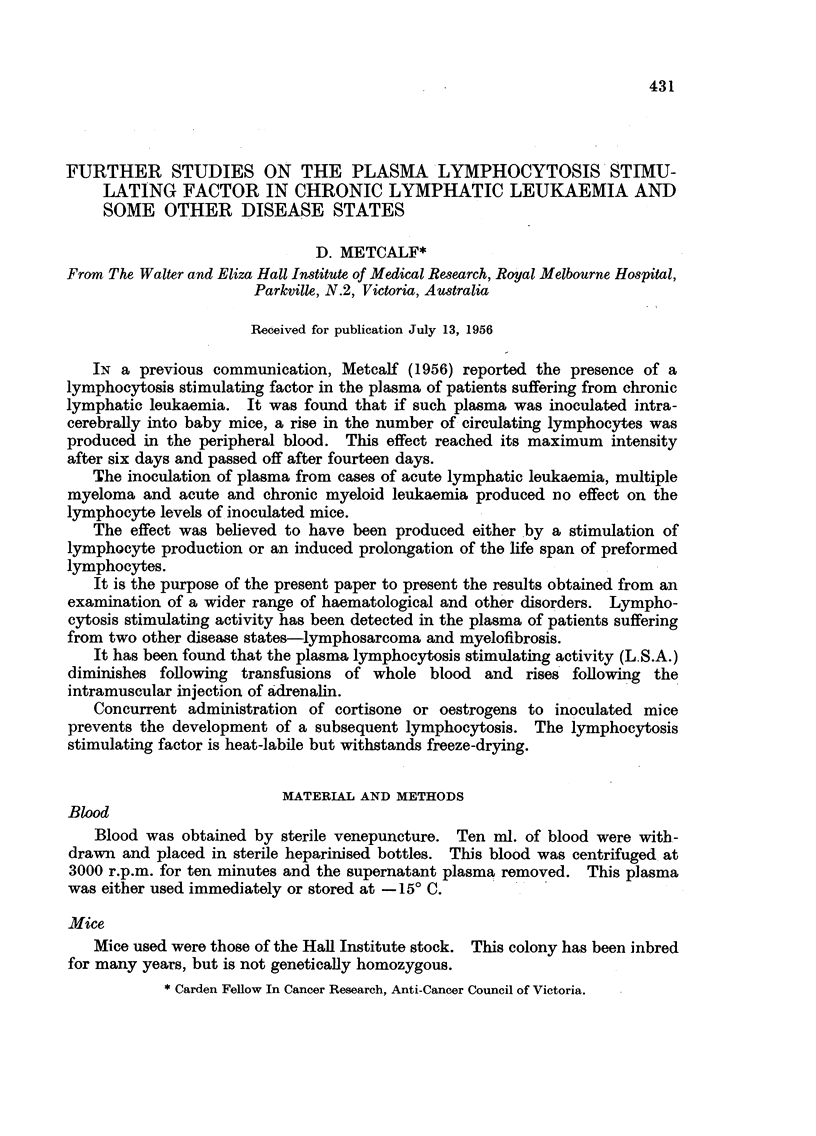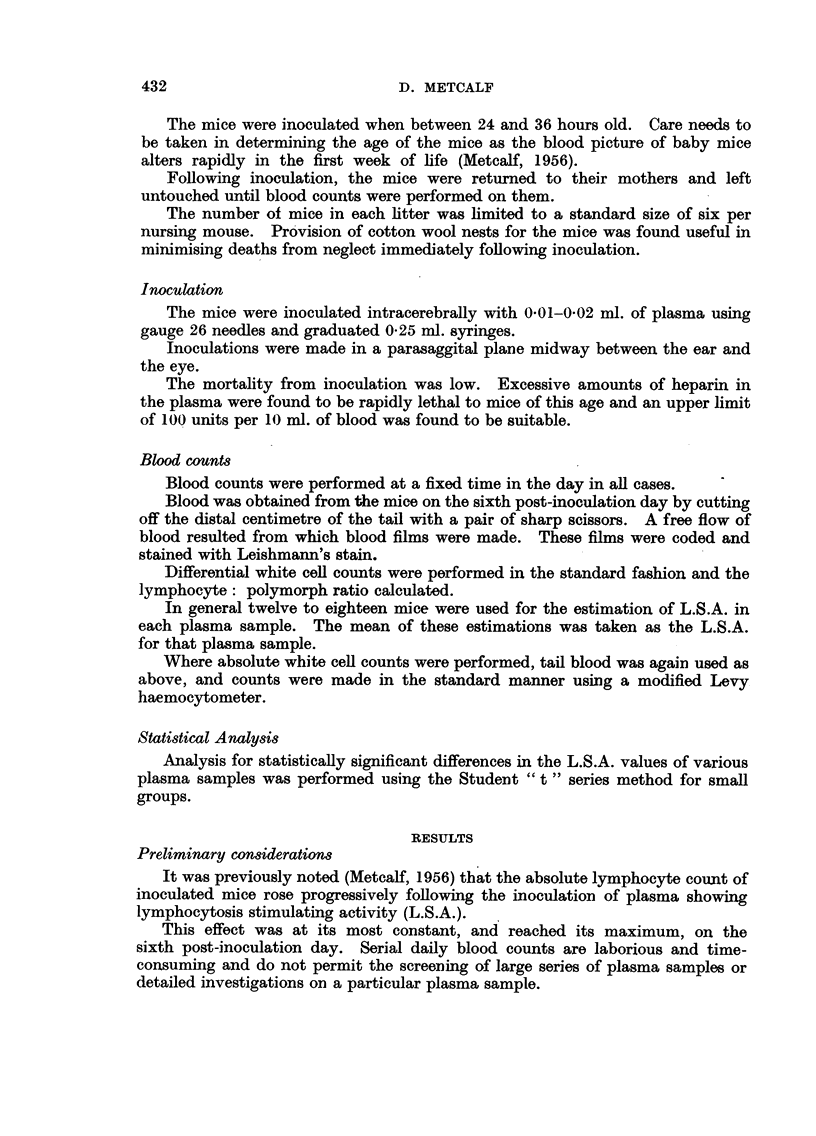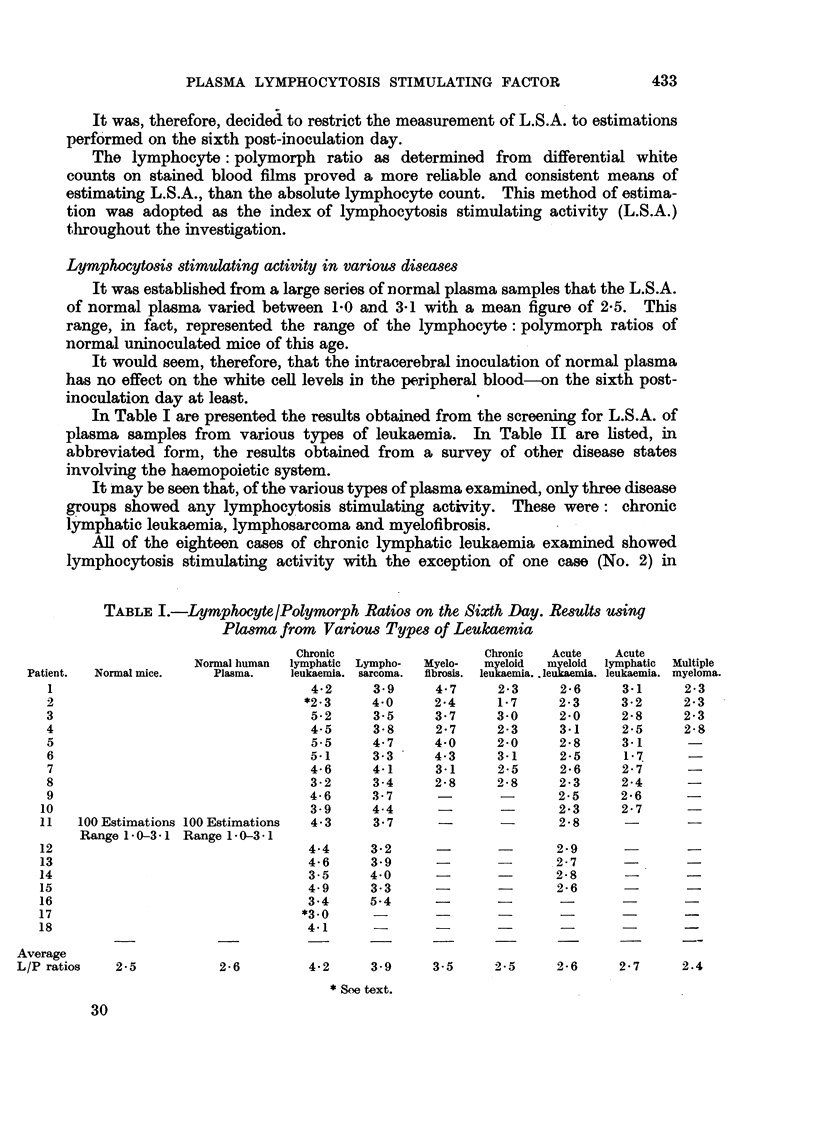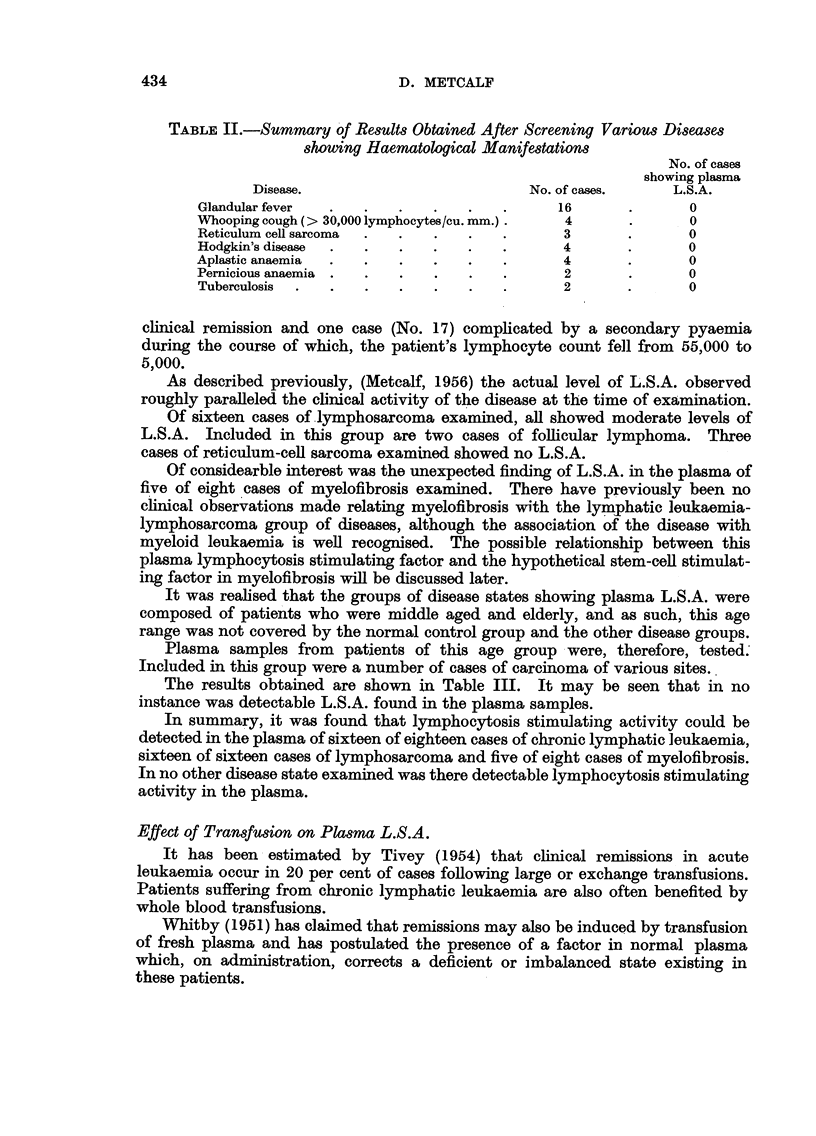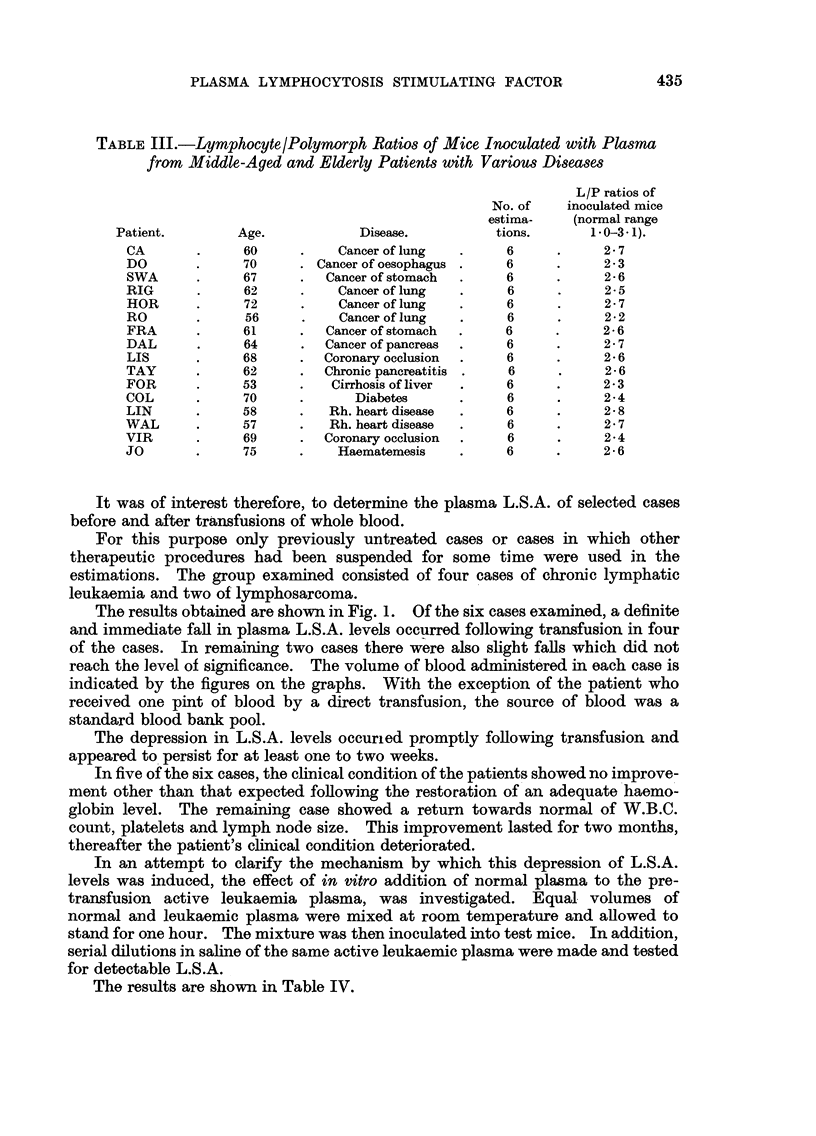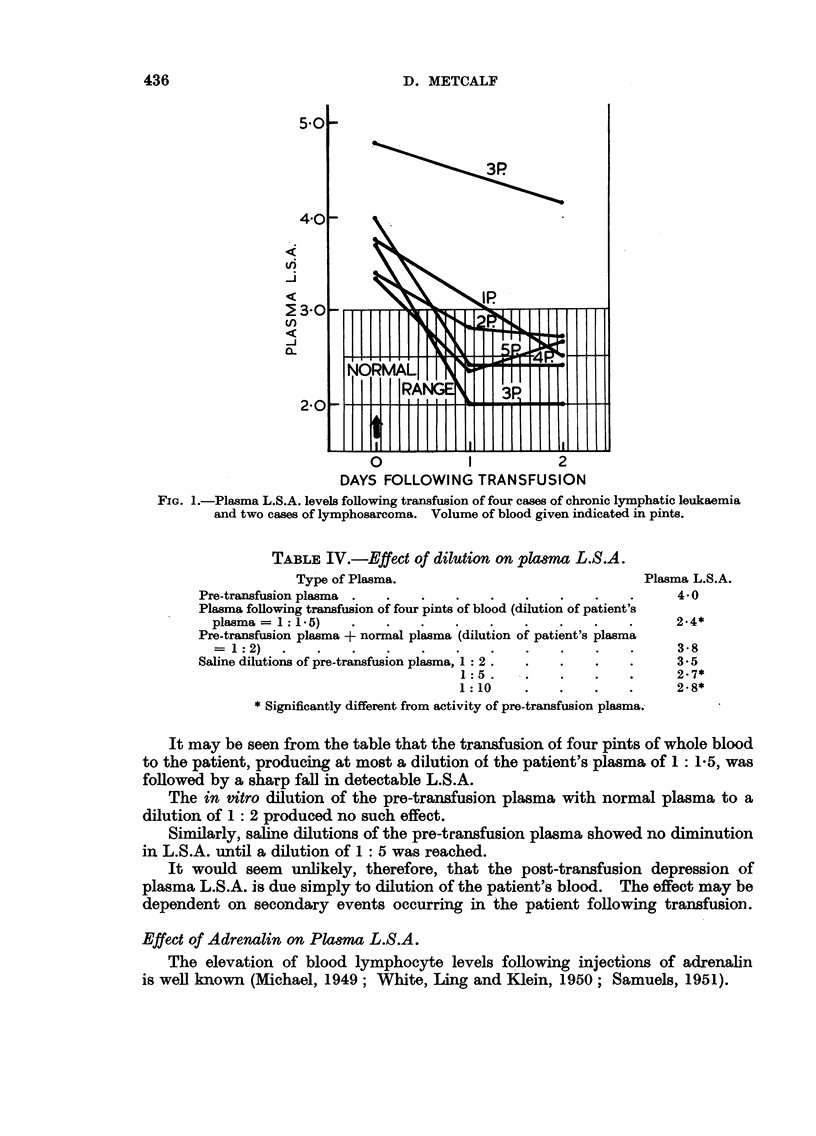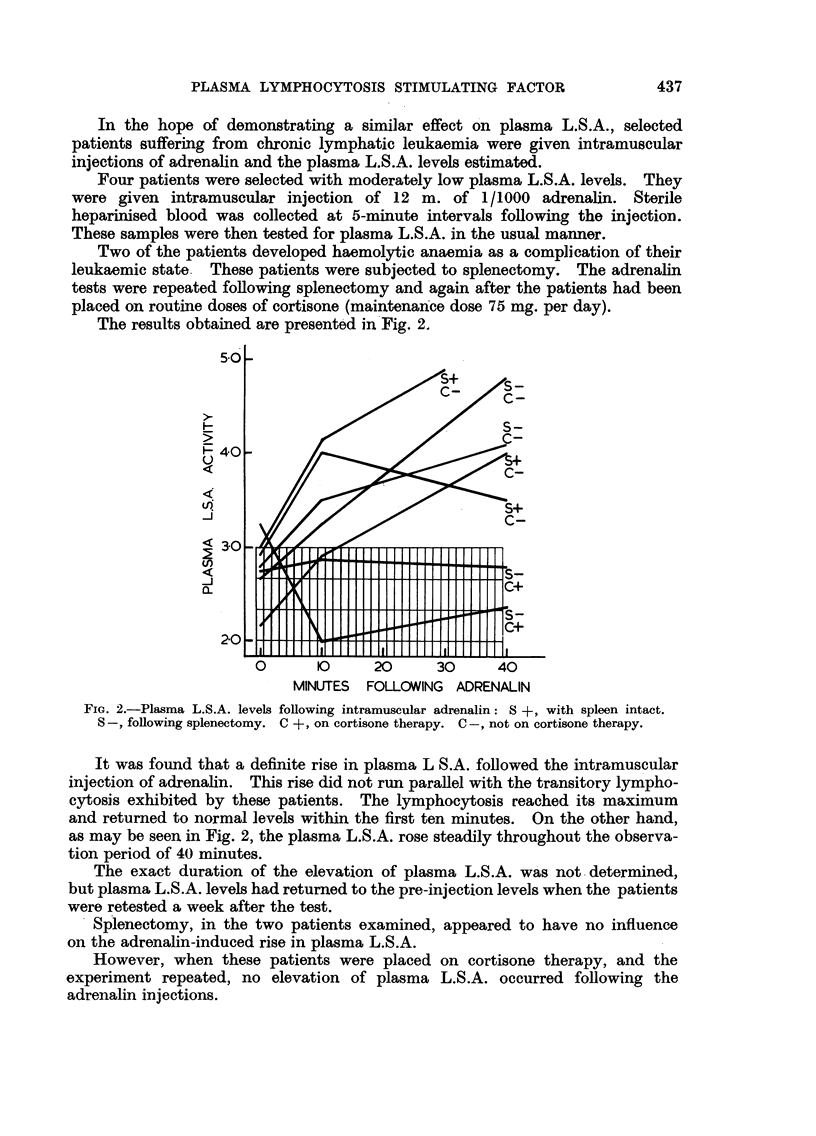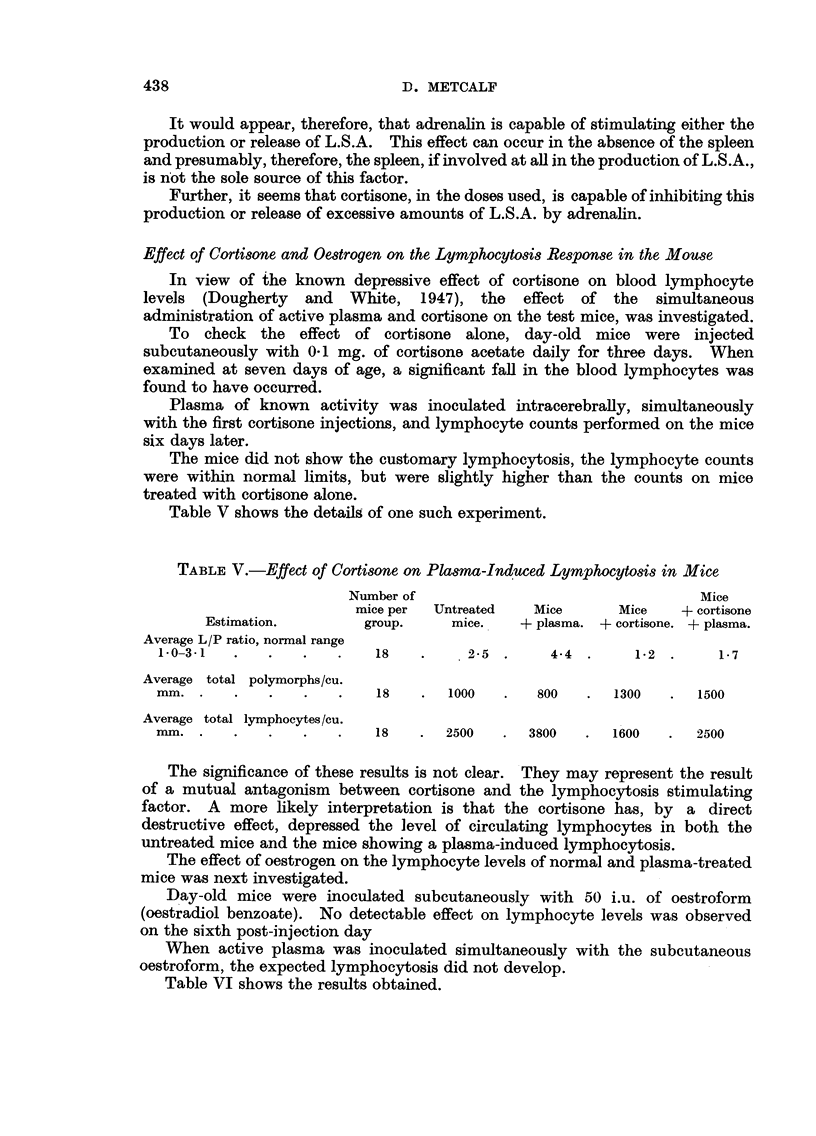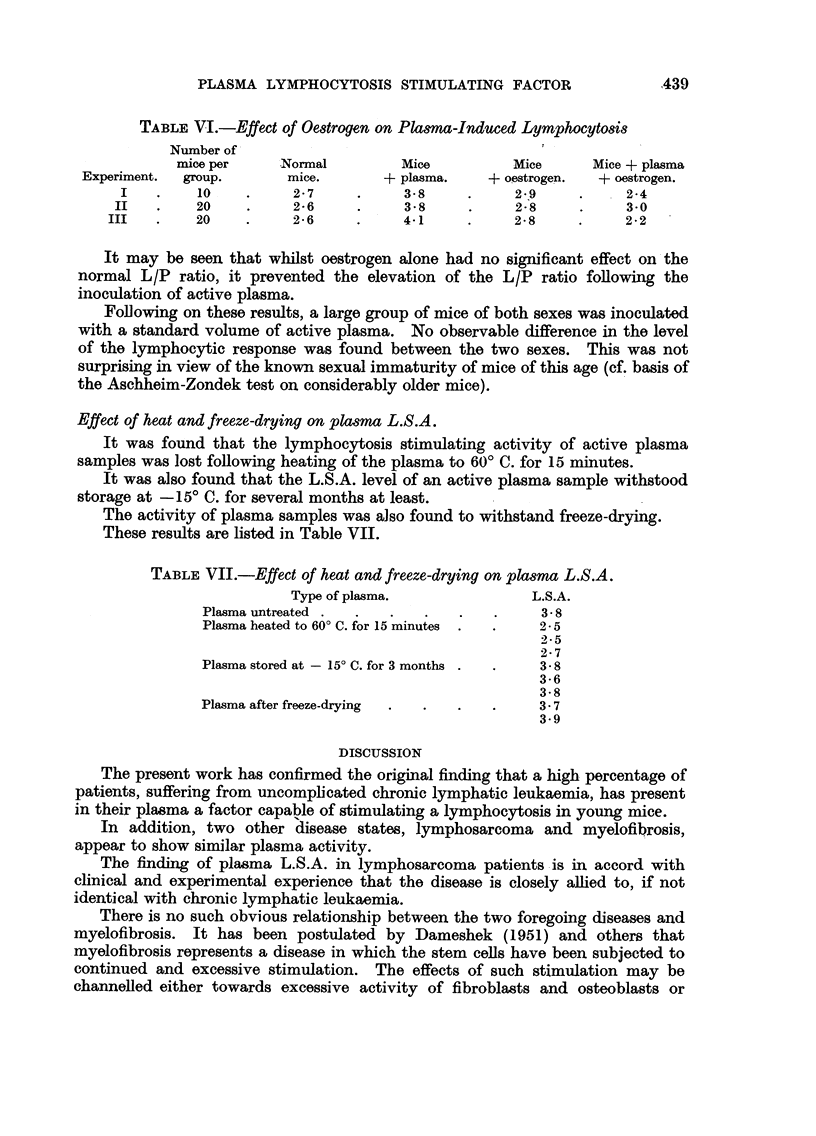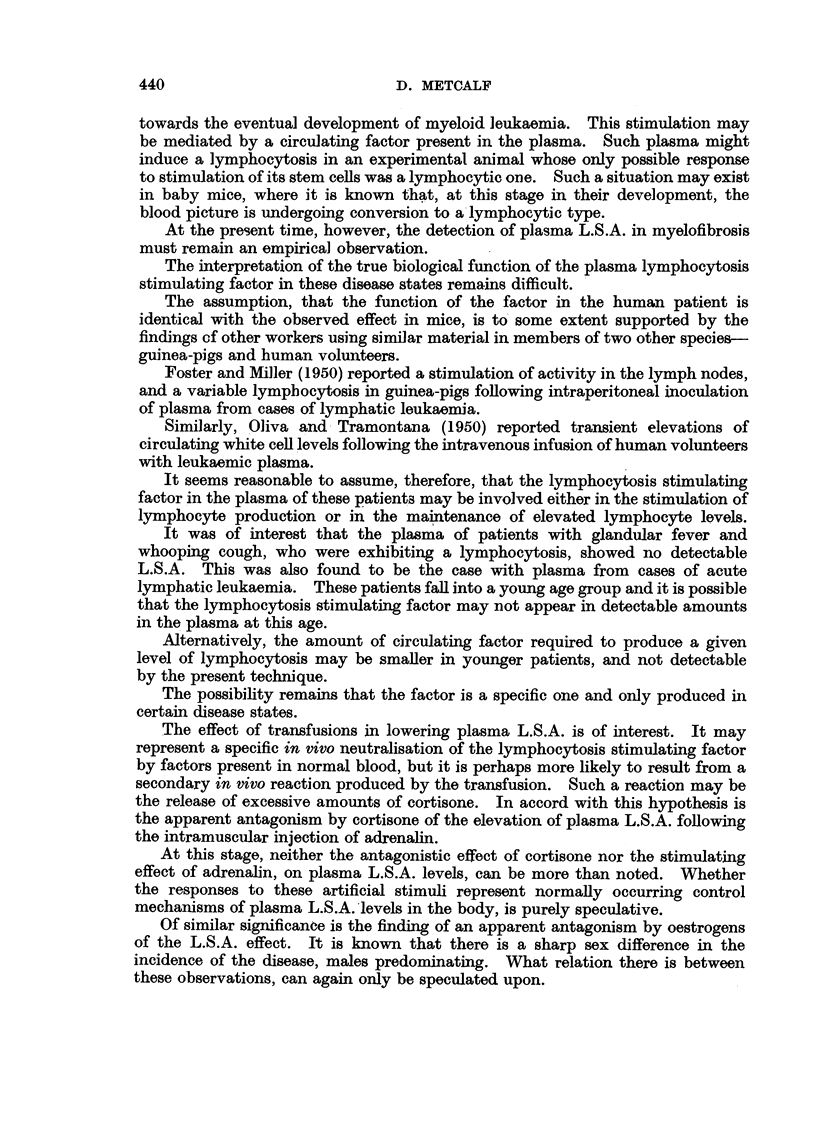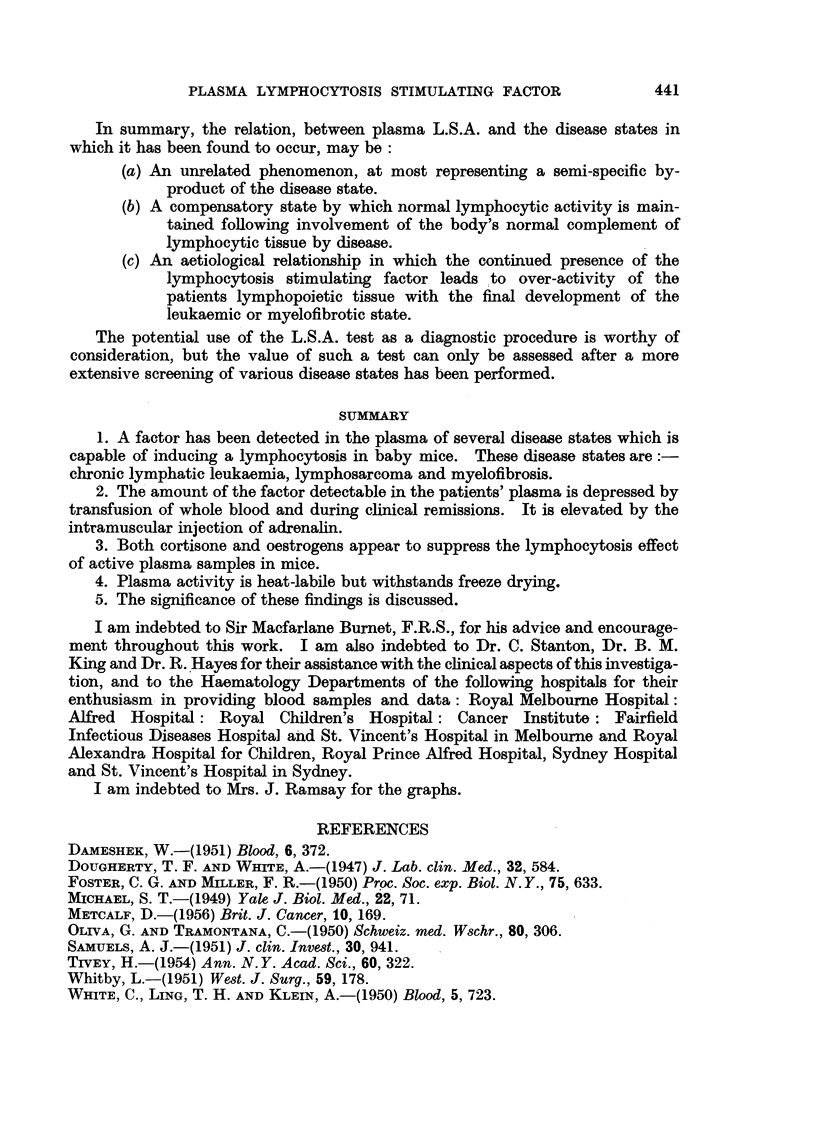# Further Studies on the Plasma Lymphocytosis Stimulating Factor in Chronic Lymphatic Leukaemia and some other Disease States

**DOI:** 10.1038/bjc.1956.50

**Published:** 1956-09

**Authors:** D. Metcalf


### FURTHER STUDIES ON THE PLASMA LYMPHOCYTOSIS STIMULATING FACTOR IN CHRONIC LYMPHATIC LEUKAEMIA AND SOME OTHER DISEASE STATES

D. METCALF*

*From The Walter and Eliza Hall Institute of Medical Research, Royal Melbourne Hospital, Parkville, N.2, Victoria, Australia*

Received for publication July 13, 1956

IN a previous communication, Metcalf (1956) reported the presence of a lymphocytosis stimulating factor in the plasma of patients suffering from chronic lymphatic leukaemia. It was found that if such plasma was inoculated intracerebrally into baby mice, a rise in the number of circulating lymphocytes was produced in the peripheral blood. This effect reached its maximum intensity after six days and passed off after fourteen days.

The inoculation of plasma from cases of acute lymphatic leukaemia, multiple myeloma and acute and chronic myeloid leukaemia produced no effect on the lymphocyte levels of inoculated mice.

The effect was believed to have been produced either by a stimulation of lymphocyte production or an induced prolongation of the life span of preformed lymphocytes.

It is the purpose of the present paper to present the results obtained from an examination of a wider range of haematological and other disorders. Lymphocytosis stimulating activity has been detected in the plasma of patients suffering from two other disease states—lymphosarcoma and myelofibrosis.

It has been found that the plasma lymphocytosis stimulating activity (L.S.A.) diminishes following transfusions of whole blood and rises following the intramuscular injection of adrenalin.

Concurrent administration of cortisone or oestrogens to inoculated mice prevents the development of a subsequent lymphocytosis. The lymphocytosis stimulating factor is heat-labile but withstands freeze-drying.

### MATERIAL AND METHODS

### *Blood*

Blood was obtained by sterile venepuncture. Ten ml. of blood were withdrawn and placed in sterile heparinised bottles. This blood was centrifuged at 3000 r.p.m. for ten minutes and the supernatant plasma removed. This plasma was either used immediately or stored at $-15°$ C.

### *Mice*

Mice used were those of the Hall Institute stock. This colony has been inbred for many years, but is not genetically homozygous.

* Carden Fellow In Cancer Research, Anti-Cancer Council of Victoria.

The mice were inoculated when between 24 and 36 hours old. Care needs to be taken in determining the age of the mice as the blood picture of baby mice alters rapidly in the first week of life (Metcalf, 1956).

Following inoculation, the mice were returned to their mothers and left untouched until blood counts were performed on them.

The number of mice in each litter was limited to a standard size of six per nursing mouse. Provision of cotton wool nests for the mice was found useful in minimising deaths from neglect immediately following inoculation.

*Inoculation*

The mice were inoculated intracerebrally with 0·01–0·02 ml. of plasma using gauge 26 needles and graduated 0·25 ml. syringes.

Inoculations were made in a parasaggital plane midway between the ear and the eye.

The mortality from inoculation was low. Excessive amounts of heparin in the plasma were found to be rapidly lethal to mice of this age and an upper limit of 100 units per 10 ml. of blood was found to be suitable.

*Blood counts*

Blood counts were performed at a fixed time in the day in all cases.

Blood was obtained from the mice on the sixth post-inoculation day by cutting off the distal centimetre of the tail with a pair of sharp scissors. A free flow of blood resulted from which blood films were made. These films were coded and stained with Leishmann's stain.

Differential white cell counts were performed in the standard fashion and the lymphocyte : polymorph ratio calculated.

In general twelve to eighteen mice were used for the estimation of L.S.A. in each plasma sample. The mean of these estimations was taken as the L.S.A. for that plasma sample.

Where absolute white cell counts were performed, tail blood was again used as above, and counts were made in the standard manner using a modified Levy haemocytometer.

*Statistical Analysis*

Analysis for statistically significant differences in the L.S.A. values of various plasma samples was performed using the Student " t " series method for small groups.

### RESULTS

*Preliminary considerations*

It was previously noted (Metcalf, 1956) that the absolute lymphocyte count of inoculated mice rose progressively following the inoculation of plasma showing lymphocytosis stimulating activity (L.S.A.).

This effect was at its most constant, and reached its maximum, on the sixth post-inoculation day. Serial daily blood counts are laborious and time-consuming and do not permit the screening of large series of plasma samples or detailed investigations on a particular plasma sample.

It was, therefore, decided to restrict the measurement of L.S.A. to estimations performed on the sixth post-inoculation day.

The lymphocyte : polymorph ratio as determined from differential white counts on stained blood films proved a more reliable and consistent means of estimating L.S.A., than the absolute lymphocyte count. This method of estimation was adopted as the index of lymphocytosis stimulating activity (L.S.A.) throughout the investigation.

*Lymphocytosis stimulating activity in various diseases*

It was established from a large series of normal plasma samples that the L.S.A. of normal plasma varied between 1·0 and 3·1 with a mean figure of 2·5. This range, in fact, represented the range of the lymphocyte : polymorph ratios of normal uninoculated mice of this age.

It would seem, therefore, that the intracerebral inoculation of normal plasma has no effect on the white cell levels in the peripheral blood—on the sixth post-inoculation day at least.

In Table I are presented the results obtained from the screening for L.S.A. of plasma samples from various types of leukaemia. In Table II are listed, in abbreviated form, the results obtained from a survey of other disease states involving the haemopoietic system.

It may be seen that, of the various types of plasma examined, only three disease groups showed any lymphocytosis stimulating activity. These were : chronic lymphatic leukaemia, lymphosarcoma and myelofibrosis.

All of the eighteen cases of chronic lymphatic leukaemia examined showed lymphocytosis stimulating activity with the exception of one case (No. 2) in

TABLE I.—*Lymphocyte/Polymorph Ratios on the Sixth Day. Results using Plasma from Various Types of Leukaemia*

| Patient. | Normal mice. | Normal human Plasma. | Chronic lymphatic leukaemia. | Lympho-sarcoma. | Myelo-fibrosis. | Chronic myeloid leukaemia. | Acute myeloid leukaemia. | Acute lymphatic leukaemia. | Multiple myeloma. |
|---|---|---|---|---|---|---|---|---|---|
| 1 | | | 4·2 | 3·9 | 4·7 | 2·3 | 2·6 | 3·1 | 2·3 |
| 2 | | | *2·3 | 4·0 | 2·4 | 1·7 | 2·3 | 3·2 | 2·3 |
| 3 | | | 5·2 | 3·5 | 3·7 | 3·0 | 2·0 | 2·8 | 2·3 |
| 4 | | | 4·5 | 3·8 | 2·7 | 2·3 | 3·1 | 2·5 | 2·8 |
| 5 | | | 5·5 | 4·7 | 4·0 | 2·0 | 2·8 | 3·1 | — |
| 6 | | | 5·1 | 3·3 | 4·3 | 3·1 | 2·5 | 1·7 | — |
| 7 | | | 4·6 | 4·1 | 3·1 | 2·5 | 2·6 | 2·7 | — |
| 8 | | | 3·2 | 3·4 | 2·8 | 2·8 | 2·3 | 2·4 | — |
| 9 | | | 4·6 | 3·7 | — | — | 2·5 | 2·6 | — |
| 10 | | | 3·9 | 4·4 | — | — | 2·3 | 2·7 | — |
| 11 | 100 Estimations Range 1·0–3·1 | 100 Estimations Range 1·0–3·1 | 4·3 | 3·7 | — | — | 2·8 | — | — |
| 12 | | | 4·4 | 3·2 | — | — | 2·9 | — | — |
| 13 | | | 4·6 | 3·9 | — | — | 2·7 | — | — |
| 14 | | | 3·5 | 4·0 | — | — | 2·8 | — | — |
| 15 | | | 4·9 | 3·3 | — | — | 2·6 | — | — |
| 16 | | | 3·4 | 5·4 | — | — | — | — | — |
| 17 | | | *3·0 | — | — | — | — | — | — |
| 18 | | | 4·1 | — | — | — | — | — | — |
| Average L/P ratios | 2·5 | 2·6 | 4·2 | 3·9 | 3·5 | 2·5 | 2·6 | 2·7 | 2·4 |

* See text.

TABLE II.—*Summary of Results Obtained After Screening Various Diseases showing Haematological Manifestations*

| Disease. | No. of cases. | No. of cases showing plasma L.S.A. |
|---|---|---|
| Glandular fever | 16 | 0 |
| Whooping cough (> 30,000 lymphocytes/cu. mm.) | 4 | 0 |
| Reticulum cell sarcoma | 3 | 0 |
| Hodgkin's disease | 4 | 0 |
| Aplastic anaemia | 4 | 0 |
| Pernicious anaemia | 2 | 0 |
| Tuberculosis | 2 | 0 |

clinical remission and one case (No. 17) complicated by a secondary pyaemia during the course of which, the patient's lymphocyte count fell from 55,000 to 5,000.

As described previously, (Metcalf, 1956) the actual level of L.S.A. observed roughly paralleled the clinical activity of the disease at the time of examination.

Of sixteen cases of lymphosarcoma examined, all showed moderate levels of L.S.A. Included in this group are two cases of follicular lymphoma. Three cases of reticulum-cell sarcoma examined showed no L.S.A.

Of considearble interest was the unexpected finding of L.S.A. in the plasma of five of eight cases of myelofibrosis examined. There have previously been no clinical observations made relating myelofibrosis with the lymphatic leukaemia-lymphosarcoma group of diseases, although the association of the disease with myeloid leukaemia is well recognised. The possible relationship between this plasma lymphocytosis stimulating factor and the hypothetical stem-cell stimulating factor in myelofibrosis will be discussed later.

It was realised that the groups of disease states showing plasma L.S.A. were composed of patients who were middle aged and elderly, and as such, this age range was not covered by the normal control group and the other disease groups.

Plasma samples from patients of this age group were, therefore, tested. Included in this group were a number of cases of carcinoma of various sites.

The results obtained are shown in Table III. It may be seen that in no instance was detectable L.S.A. found in the plasma samples.

In summary, it was found that lymphocytosis stimulating activity could be detected in the plasma of sixteen of eighteen cases of chronic lymphatic leukaemia, sixteen of sixteen cases of lymphosarcoma and five of eight cases of myelofibrosis. In no other disease state examined was there detectable lymphocytosis stimulating activity in the plasma.

*Effect of Transfusion on Plasma L.S.A.*

It has been estimated by Tivey (1954) that clinical remissions in acute leukaemia occur in 20 per cent of cases following large or exchange transfusions. Patients suffering from chronic lymphatic leukaemia are also often benefited by whole blood transfusions.

Whitby (1951) has claimed that remissions may also be induced by transfusion of fresh plasma and has postulated the presence of a factor in normal plasma which, on administration, corrects a deficient or imbalanced state existing in these patients.

TABLE III.—*Lymphocyte/Polymorph Ratios of Mice Inoculated with Plasma from Middle-Aged and Elderly Patients with Various Diseases*

| Patient. | Age. | Disease. | No. of estimations. | L/P ratios of inoculated mice (normal range 1·0–3·1). |
|---|---|---|---|---|
| CA | 60 | Cancer of lung | 6 | 2·7 |
| DO | 70 | Cancer of oesophagus | 6 | 2·3 |
| SWA | 67 | Cancer of stomach | 6 | 2·6 |
| RIG | 62 | Cancer of lung | 6 | 2·5 |
| HOR | 72 | Cancer of lung | 6 | 2·7 |
| RO | 56 | Cancer of lung | 6 | 2·2 |
| FRA | 61 | Cancer of stomach | 6 | 2·6 |
| DAL | 64 | Cancer of pancreas | 6 | 2·7 |
| LIS | 68 | Coronary occlusion | 6 | 2·6 |
| TAY | 62 | Chronic pancreatitis | 6 | 2·6 |
| FOR | 53 | Cirrhosis of liver | 6 | 2·3 |
| COL | 70 | Diabetes | 6 | 2·4 |
| LIN | 58 | Rh. heart disease | 6 | 2·8 |
| WAL | 57 | Rh. heart disease | 6 | 2·7 |
| VIR | 69 | Coronary occlusion | 6 | 2·4 |
| JO | 75 | Haematemesis | 6 | 2·6 |

It was of interest therefore, to determine the plasma L.S.A. of selected cases before and after transfusions of whole blood.

For this purpose only previously untreated cases or cases in which other therapeutic procedures had been suspended for some time were used in the estimations. The group examined consisted of four cases of chronic lymphatic leukaemia and two of lymphosarcoma.

The results obtained are shown in Fig. 1. Of the six cases examined, a definite and immediate fall in plasma L.S.A. levels occurred following transfusion in four of the cases. In remaining two cases there were also slight falls which did not reach the level of significance. The volume of blood administered in each case is indicated by the figures on the graphs. With the exception of the patient who received one pint of blood by a direct transfusion, the source of blood was a standard blood bank pool.

The depression in L.S.A. levels occurred promptly following transfusion and appeared to persist for at least one to two weeks.

In five of the six cases, the clinical condition of the patients showed no improvement other than that expected following the restoration of an adequate haemoglobin level. The remaining case showed a return towards normal of W.B.C. count, platelets and lymph node size. This improvement lasted for two months, thereafter the patient's clinical condition deteriorated.

In an attempt to clarify the mechanism by which this depression of L.S.A. levels was induced, the effect of *in vitro* addition of normal plasma to the pre-transfusion active leukaemia plasma, was investigated. Equal volumes of normal and leukaemic plasma were mixed at room temperature and allowed to stand for one hour. The mixture was then inoculated into test mice. In addition, serial dilutions in saline of the same active leukaemic plasma were made and tested for detectable L.S.A.

The results are shown in Table IV.

FIG. 1.—Plasma L.S.A. levels following transfusion of four cases of chronic lymphatic leukaemia and two cases of lymphosarcoma. Volume of blood given indicated in pints.

TABLE IV.—*Effect of dilution on plasma L.S.A.*

| Type of Plasma. | Plasma L.S.A. |
|---|---|
| Pre-transfusion plasma . . . . . . . . . . . | 4·0 |
| Plasma following transfusion of four pints of blood (dilution of patient's plasma = 1 : 1·5) . . . . . . . . . . . | 2·4* |
| Pre-transfusion plasma + normal plasma (dilution of patient's plasma = 1 : 2) . . . . . . . . . . . . . | 3·8 |
| Saline dilutions of pre-transfusion plasma, 1 : 2 . . . . . . | 3·5 |
| 1 : 5 . . . . . | 2·7* |
| 1 : 10 . . . . | 2·8* |

* Significantly different from activity of pre-transfusion plasma.

It may be seen from the table that the transfusion of four pints of whole blood to the patient, producing at most a dilution of the patient's plasma of 1 : 1·5, was followed by a sharp fall in detectable L.S.A.

The *in vitro* dilution of the pre-transfusion plasma with normal plasma to a dilution of 1 : 2 produced no such effect.

Similarly, saline dilutions of the pre-transfusion plasma showed no diminution in L.S.A. until a dilution of 1 : 5 was reached.

It would seem unlikely, therefore, that the post-transfusion depression of plasma L.S.A. is due simply to dilution of the patient's blood. The effect may be dependent on secondary events occurring in the patient following transfusion.

### *Effect of Adrenalin on Plasma L.S.A.*

The elevation of blood lymphocyte levels following injections of adrenalin is well known (Michael, 1949 ; White, Ling and Klein, 1950 ; Samuels, 1951).

In the hope of demonstrating a similar effect on plasma L.S.A., selected patients suffering from chronic lymphatic leukaemia were given intramuscular injections of adrenalin and the plasma L.S.A. levels estimated.

Four patients were selected with moderately low plasma L.S.A. levels. They were given intramuscular injection of 12 m. of 1/1000 adrenalin. Sterile heparinised blood was collected at 5-minute intervals following the injection. These samples were then tested for plasma L.S.A. in the usual manner.

Two of the patients developed haemolytic anaemia as a complication of their leukaemic state. These patients were subjected to splenectomy. The adrenalin tests were repeated following splenectomy and again after the patients had been placed on routine doses of cortisone (maintenance dose 75 mg. per day).

The results obtained are presented in Fig. 2.

FIG. 2.—Plasma L.S.A. levels following intramuscular adrenalin: S +, with spleen intact. S —, following splenectomy. C +, on cortisone therapy. C —, not on cortisone therapy.

It was found that a definite rise in plasma L S.A. followed the intramuscular injection of adrenalin. This rise did not run parallel with the transitory lymphocytosis exhibited by these patients. The lymphocytosis reached its maximum and returned to normal levels within the first ten minutes. On the other hand, as may be seen in Fig. 2, the plasma L.S.A. rose steadily throughout the observation period of 40 minutes.

The exact duration of the elevation of plasma L.S.A. was not determined, but plasma L.S.A. levels had returned to the pre-injection levels when the patients were retested a week after the test.

Splenectomy, in the two patients examined, appeared to have no influence on the adrenalin-induced rise in plasma L.S.A.

However, when these patients were placed on cortisone therapy, and the experiment repeated, no elevation of plasma L.S.A. occurred following the adrenalin injections.

It would appear, therefore, that adrenalin is capable of stimulating either the production or release of L.S.A. This effect can occur in the absence of the spleen and presumably, therefore, the spleen, if involved at all in the production of L.S.A., is not the sole source of this factor.

Further, it seems that cortisone, in the doses used, is capable of inhibiting this production or release of excessive amounts of L.S.A. by adrenalin.

*Effect of Cortisone and Oestrogen on the Lymphocytosis Response in the Mouse*

In view of the known depressive effect of cortisone on blood lymphocyte levels (Dougherty and White, 1947), the effect of the simultaneous administration of active plasma and cortisone on the test mice, was investigated.

To check the effect of cortisone alone, day-old mice were injected subcutaneously with 0·1 mg. of cortisone acetate daily for three days. When examined at seven days of age, a significant fall in the blood lymphocytes was found to have occurred.

Plasma of known activity was inoculated intracerebrally, simultaneously with the first cortisone injections, and lymphocyte counts performed on the mice six days later.

The mice did not show the customary lymphocytosis, the lymphocyte counts were within normal limits, but were slightly higher than the counts on mice treated with cortisone alone.

Table V shows the details of one such experiment.

TABLE V.—*Effect of Cortisone on Plasma-Induced Lymphocytosis in Mice*

| Estimation. | Number of mice per group. | Untreated mice. | Mice + plasma. | Mice + cortisone. | Mice + cortisone + plasma. |
|---|---|---|---|---|---|
| Average L/P ratio, normal range 1·0–3·1 | 18 | 2·5 | 4·4 | 1·2 | 1·7 |
| Average total polymorphs/cu. mm. | 18 | 1000 | 800 | 1300 | 1500 |
| Average total lymphocytes/cu. mm. | 18 | 2500 | 3800 | 1600 | 2500 |

The significance of these results is not clear. They may represent the result of a mutual antagonism between cortisone and the lymphocytosis stimulating factor. A more likely interpretation is that the cortisone has, by a direct destructive effect, depressed the level of circulating lymphocytes in both the untreated mice and the mice showing a plasma-induced lymphocytosis.

The effect of oestrogen on the lymphocyte levels of normal and plasma-treated mice was next investigated.

Day-old mice were inoculated subcutaneously with 50 i.u. of oestroform (oestradiol benzoate). No detectable effect on lymphocyte levels was observed on the sixth post-injection day

When active plasma was inoculated simultaneously with the subcutaneous oestroform, the expected lymphocytosis did not develop.

Table VI shows the results obtained.

TABLE VI.—*Effect of Oestrogen on Plasma-Induced Lymphocytosis*

| Experiment. | Number of mice per group. | Normal mice. | Mice + plasma. | Mice + oestrogen. | Mice + plasma + oestrogen. |
|---|---|---|---|---|---|
| I | 10 | 2·7 | 3·8 | 2·9 | 2·4 |
| II | 20 | 2·6 | 3·8 | 2·8 | 3·0 |
| III | 20 | 2·6 | 4·1 | 2·8 | 2·2 |

It may be seen that whilst oestrogen alone had no significant effect on the normal L/P ratio, it prevented the elevation of the L/P ratio following the inoculation of active plasma.

Following on these results, a large group of mice of both sexes was inoculated with a standard volume of active plasma. No observable difference in the level of the lymphocytic response was found between the two sexes. This was not surprising in view of the known sexual immaturity of mice of this age (cf. basis of the Aschheim-Zondek test on considerably older mice).

*Effect of heat and freeze-drying on plasma L.S.A.*

It was found that the lymphocytosis stimulating activity of active plasma samples was lost following heating of the plasma to 60° C. for 15 minutes.

It was also found that the L.S.A. level of an active plasma sample withstood storage at —15° C. for several months at least.

The activity of plasma samples was also found to withstand freeze-drying. These results are listed in Table VII.

TABLE VII.—*Effect of heat and freeze-drying on plasma L.S.A.*

| Type of plasma. | L.S.A. |
|---|---|
| Plasma untreated | 3·8 |
| Plasma heated to 60° C. for 15 minutes | 2·5 |
| | 2·5 |
| | 2·7 |
| Plasma stored at — 15° C. for 3 months | 3·8 |
| | 3·6 |
| | 3·8 |
| Plasma after freeze-drying | 3·7 |
| | 3·9 |

### DISCUSSION

The present work has confirmed the original finding that a high percentage of patients, suffering from uncomplicated chronic lymphatic leukaemia, has present in their plasma a factor capable of stimulating a lymphocytosis in young mice.

In addition, two other disease states, lymphosarcoma and myelofibrosis, appear to show similar plasma activity.

The finding of plasma L.S.A. in lymphosarcoma patients is in accord with clinical and experimental experience that the disease is closely allied to, if not identical with chronic lymphatic leukaemia.

There is no such obvious relationship between the two foregoing diseases and myelofibrosis. It has been postulated by Dameshek (1951) and others that myelofibrosis represents a disease in which the stem cells have been subjected to continued and excessive stimulation. The effects of such stimulation may be channelled either towards excessive activity of fibroblasts and osteoblasts or towards the eventual development of myeloid leukaemia. This stimulation may be mediated by a circulating factor present in the plasma. Such plasma might induce a lymphocytosis in an experimental animal whose only possible response to stimulation of its stem cells was a lymphocytic one. Such a situation may exist in baby mice, where it is known that, at this stage in their development, the blood picture is undergoing conversion to a lymphocytic type.

At the present time, however, the detection of plasma L.S.A. in myelofibrosis must remain an empirical observation.

The interpretation of the true biological function of the plasma lymphocytosis stimulating factor in these disease states remains difficult.

The assumption, that the function of the factor in the human patient is identical with the observed effect in mice, is to some extent supported by the findings of other workers using similar material in members of two other species—guinea-pigs and human volunteers.

Foster and Miller (1950) reported a stimulation of activity in the lymph nodes, and a variable lymphocytosis in guinea-pigs following intraperitoneal inoculation of plasma from cases of lymphatic leukaemia.

Similarly, Oliva and Tramontana (1950) reported transient elevations of circulating white cell levels following the intravenous infusion of human volunteers with leukaemic plasma.

It seems reasonable to assume, therefore, that the lymphocytosis stimulating factor in the plasma of these patients may be involved either in the stimulation of lymphocyte production or in the maintenance of elevated lymphocyte levels.

It was of interest that the plasma of patients with glandular fever and whooping cough, who were exhibiting a lymphocytosis, showed no detectable L.S.A. This was also found to be the case with plasma from cases of acute lymphatic leukaemia. These patients fall into a young age group and it is possible that the lymphocytosis stimulating factor may not appear in detectable amounts in the plasma at this age.

Alternatively, the amount of circulating factor required to produce a given level of lymphocytosis may be smaller in younger patients, and not detectable by the present technique.

The possibility remains that the factor is a specific one and only produced in certain disease states.

The effect of transfusions in lowering plasma L.S.A. is of interest. It may represent a specific *in vivo* neutralisation of the lymphocytosis stimulating factor by factors present in normal blood, but it is perhaps more likely to result from a secondary *in vivo* reaction produced by the transfusion. Such a reaction may be the release of excessive amounts of cortisone. In accord with this hypothesis is the apparent antagonism by cortisone of the elevation of plasma L.S.A. following the intramuscular injection of adrenalin.

At this stage, neither the antagonistic effect of cortisone nor the stimulating effect of adrenalin, on plasma L.S.A. levels, can be more than noted. Whether the responses to these artificial stimuli represent normally occurring control mechanisms of plasma L.S.A. levels in the body, is purely speculative.

Of similar significance is the finding of an apparent antagonism by oestrogens of the L.S.A. effect. It is known that there is a sharp sex difference in the incidence of the disease, males predominating. What relation there is between these observations, can again only be speculated upon.

In summary, the relation, between plasma L.S.A. and the disease states in which it has been found to occur, may be :

(*a*) An unrelated phenomenon, at most representing a semi-specific by-product of the disease state.
(*b*) A compensatory state by which normal lymphocytic activity is maintained following involvement of the body's normal complement of lymphocytic tissue by disease.
(*c*) An aetiological relationship in which the continued presence of the lymphocytosis stimulating factor leads to over-activity of the patients lymphopoietic tissue with the final development of the leukaemic or myelofibrotic state.

The potential use of the L.S.A. test as a diagnostic procedure is worthy of consideration, but the value of such a test can only be assessed after a more extensive screening of various disease states has been performed.

### SUMMARY

1. A factor has been detected in the plasma of several disease states which is capable of inducing a lymphocytosis in baby mice. These disease states are :—chronic lymphatic leukaemia, lymphosarcoma and myelofibrosis.

2. The amount of the factor detectable in the patients' plasma is depressed by transfusion of whole blood and during clinical remissions. It is elevated by the intramuscular injection of adrenalin.

3. Both cortisone and oestrogens appear to suppress the lymphocytosis effect of active plasma samples in mice.

4. Plasma activity is heat-labile but withstands freeze drying.

5. The significance of these findings is discussed.

I am indebted to Sir Macfarlane Burnet, F.R.S., for his advice and encouragement throughout this work. I am also indebted to Dr. C. Stanton, Dr. B. M. King and Dr. R. Hayes for their assistance with the clinical aspects of this investigation, and to the Haematology Departments of the following hospitals for their enthusiasm in providing blood samples and data : Royal Melbourne Hospital : Alfred Hospital : Royal Children's Hospital : Cancer Institute : Fairfield Infectious Diseases Hospital and St. Vincent's Hospital in Melbourne and Royal Alexandra Hospital for Children, Royal Prince Alfred Hospital, Sydney Hospital and St. Vincent's Hospital in Sydney.

I am indebted to Mrs. J. Ramsay for the graphs.

### REFERENCES

DAMESHEK, W.—(1951) *Blood*, **6**, 372.
DOUGHERTY, T. F. AND WHITE, A.—(1947) *J. Lab. clin. Med.*, **32**, 584.
FOSTER, C. G. AND MILLER, F. R.—(1950) *Proc. Soc. exp. Biol. N.Y.*, **75**, 633.
MICHAEL, S. T.—(1949) *Yale J. Biol. Med.*, **22**, 71.
METCALF, D.—(1956) *Brit. J. Cancer*, **10**, 169.
OLIVA, G. AND TRAMONTANA, C.—(1950) *Schweiz. med. Wschr.*, **80**, 306.
SAMUELS, A. J.—(1951) *J. clin. Invest.*, **30**, 941.
TIVEY, H.—(1954) *Ann. N.Y. Acad. Sci.*, **60**, 322.
Whitby, L.—(1951) *West. J. Surg.*, **59**, 178.
WHITE, C., LING, T. H. AND KLEIN, A.—(1950) *Blood*, **5**, 723.